# Impact of Natural Juice Consumption on Plasma Antioxidant Status: A Systematic Review and Meta-Analysis

**DOI:** 10.3390/molecules201219834

**Published:** 2015-12-10

**Authors:** Fernanda S. Tonin, Laiza M. Steimbach, Astrid Wiens, Cássio M. Perlin, Roberto Pontarolo

**Affiliations:** 1 Department of Pharmacy, Pharmaceutical Sciences Postgraduate Program, Universidade Federal do Paraná, Av. Prof. Lothario Meissner 632, 80210-170 Curitiba, Brazil; fer_stumpf_tonin@hotmail.com (F.S.T.); laizasteimbach@gmail.com (L.M.S.); astridwiens@hotmail.com (A.W.); 2 Department of Pharmacy, Postgraduate Program in Pharmaceutical Care, Universidade Federal do Rio Grande do Sul, Av. Paulo Gama 110, 90040-060 Porto Alegre, Brazil; mperlin.cassio@gmail.com

**Keywords:** antioxidant capacity, juice, health benefits, systematic review

## Abstract

Background: Oxidative stress may lead to overproduction of reactive species and a decrease in antioxidant defenses, resulting in chronic diseases such as diabetes and cancer. The consumption of natural compounds with an antioxidant profile may be a preventive alternative. Therefore, we aimed to obtain evidence regarding the potential antioxidant activity of juices in human plasma. Methods: A systematic review and meta-analysis was performed, which included randomized controlled trials that compared the use of fruit or vegetable juices *vs.* placebo or other beverages. An electronic search was conducted in PubMed, Scopus, International Pharmaceutical Abstracts, and SciELO. The outcome measures extracted were related to antioxidant status, e.g., vitamin C, superoxide dismutase (SOD), and catalase (CAT) levels and reduction in malondialdehyde (MDA) and antioxidant capacity measured as TEAC. Results: Twenty-eight trials were identified (*n* = 1089), of which 16 were used for meta-analysis. No significant differences were observed between juices and placebo with regard to TEAC, SOD, and CAT. However, juices were superior to control in enhancing vitamin C and reducing MDA. Conclusions: Natural juices are possible candidates for the management of oxidative stress. The effects of juices should be further investigated by conducting larger and well-defined trials of longer duration.

## 1. Introduction

Oxidative stress is characterized by an imbalance between the production of reactive species and antioxidant defense activity, and enhancement of oxidative stress has been associated with many conditions and chronic diseases such as cancer, diabetes, pulmonary disorders, neurodegenerative (e.g., Alzheimer’s disease) and cardiovascular diseases, and aging [1,2].

Antioxidant agents prevent oxidative stress and its consequences in cells and tissues (e.g., lipid peroxidation) that culminate in diseases. An antioxidant is a molecule that is sufficiently stable to donate an electron to a rampaging free radical and neutralize it. The antioxidants delay or inhibit cellular damage mainly through their free radical scavenging property and can safely interact with free radicals and terminate the chain reaction before vital molecules are damaged. Some such antioxidants, including glutathione, ubiquinol, and uric acid, are produced during normal metabolism. Other antioxidants are obtained from the diet [1,3].

Many research groups have analyzed the antioxidant properties of natural products. These properties have been investigated through chemical or biological methods, or both. It has been suggested that the consumption of food rich in antioxidants can retard or avoid the occurrence of many diseases [2,4].

In this context, there has been growing appreciation and understanding of the link between fruit and vegetable consumption and improved health. Biologically active components in plant-based foods, such as redox-active antioxidants (polyphenols, carotenoids, tocopherols, vitamins C and E, glutathione) and enzymes (superoxide dismutase (SOD) and catalase (CAT)) with antioxidant activity have high potential for modulating many processes during the development of diseases [5,6].

Guidelines for health promotion and disease prevention in the United States and around the world include recommendations to consume a variety of fruits and vegetables each day, since they provide significant quantities of nutrients, especially vitamins, sugars, minerals, and fiber [6,7,8].

An alternative way to consume proper amounts of fruits and vegetables is to choose beverages such as juices. During the last few years, the demand for these beverages has been increasing in several countries [4]. This may be attributed to change in dietary habits, taste preferences, and the way of life of present-day consumers [9].

Although it is universally accepted that fruit and vegetable intake can provide protective antioxidant status, there is no clear consensus about the effects of juice consumption on oxidative stress in humans. Many epidemiological studies have supported their beneficial effects, but large-scale double-blind, randomized, controlled clinical intervention studies and meta-analyses have not yet confirmed this premise [10].

Therefore, we conducted a systematic review and meta-analysis of all published randomized controlled trials (RCTs) to obtain evidence and provide a more precise estimate of the effect of fruit and vegetable juices on antioxidant status in human plasma, which could support their beneficial effects.

## 2. Results and Discussion

**Figure 1:**
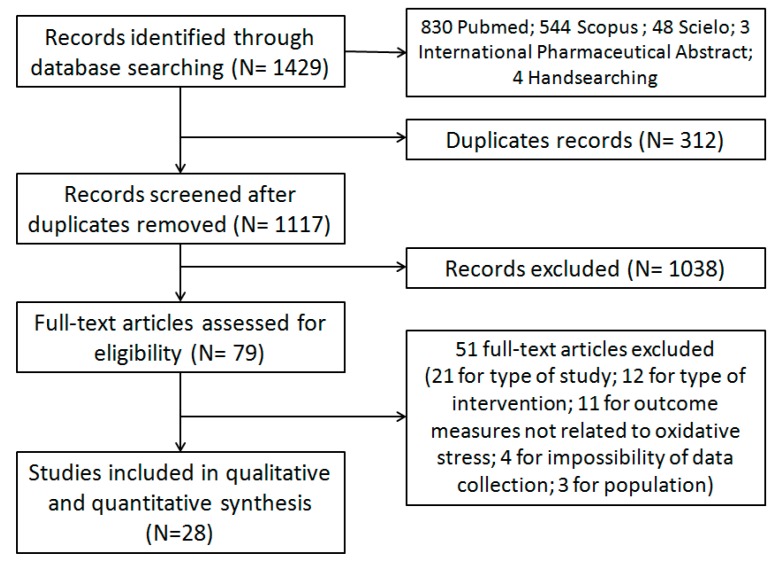
Flowchart of included Randomized Controlled Trials.

Through electronic searches, 1117 studies were retrieved after exclusion of duplicates. During the screening process (reviewing of titles and abstracts), 1038 records were excluded. After full-text analysis, 51 articles were excluded (Table S1, Appendix 1, see in Supplementary Materials); thus, 28 studies that were suitable for performing qualitative and quantitative analyses were obtained (Figure 1).

The 28 studies (*n* = 1089) included evaluated the effects of consumption of fruit or vegetable juices on healthy people or patients who had chronic diseases or a debilitating condition (e.g., diabetes, cancer, hemodialysis) [11,12,13,14,15,16,17,18,19,20,21,22,23,24,25,26,27,28,29,30,31,32,33,34,35,36,37,38]. Most studies (75%) utilized a parallel design and 17 of them used a placebo juice (mainly composed by flavored water and sucralose) for comparison. Another three studies used water as placebo and in eight studies an active control—another juice—was employed. The mean age of the subjects was 44.07 years (standard deviation 4.70) with a range between 20–70 years. The mean treatment duration was 3.6 weeks and intervention juices dosage ranged from 100 mL to 1000 mL daily. Juices compositions also varied among studies (e.g., derived from fruit and vegetables such as carrot, tomato, orange, different types of berries, grapes) (Table 1).

The quality assessment showed overall poor quality, resulting in a mean Jadad score [39] of 2.25 (range 1–4) (Table S2). All the studies scored, at least, on randomization and some trials were double-blinded. However, few studies described those methods properly and accounted for patient’s withdrawals or dropouts. Moreover, considering the risk of bias instrument, studies often failed to provide details about randomization procedures, allocation concealment, or blinding of participants and outcome assessments. More than 70% of studies (*n* = 20) were funded by industries or organization or presented conflicts of interest (Figures S2 and S3).

From the 28 eligible studies, 16 could be included in one or more meta-analysis of intervention *vs.* placebo, since they reported properly data for the outcomes measures related to antioxidant plasma activity or oxidative stress. The measures included weight gain during study (mean difference, MD from baseline); levels of the antioxidants vitamin C, SOD, and CAT; analysis of the biomarker malondialdehyde (MDA) related to lipid peroxidation and TEAC (Trolox-equivalent antioxidant capacity).

**Figure 2:**
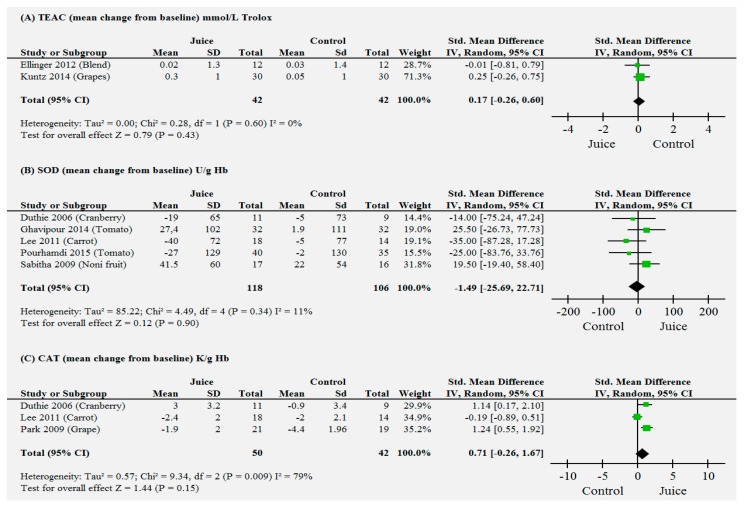

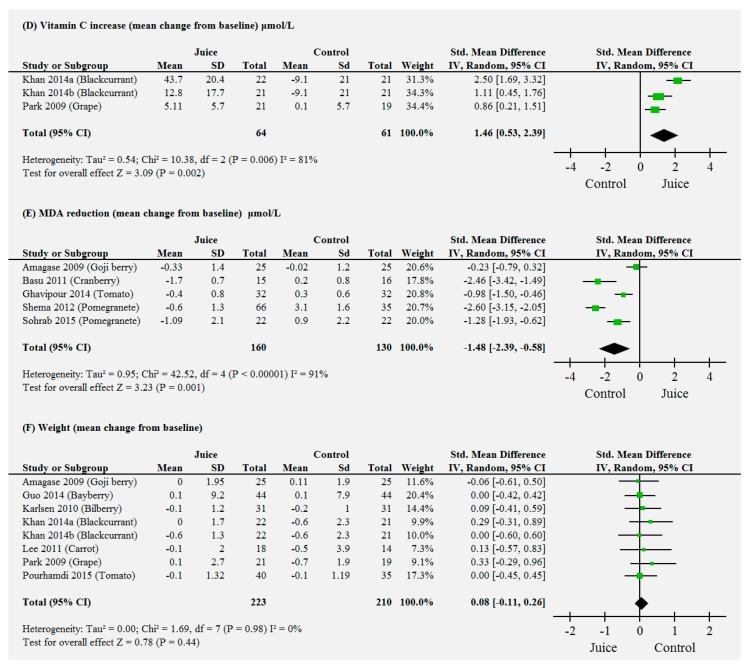
Forest plot for the outcome measures related to antioxidant status in the human plasma. Mean change from baseline for (**A**): Trolox equivalent antioxidant capacity (TEAC); (**B**) superoxide dismutase enzyme (SOD); (**C**): catalase enzyme (CAT). Statistical method: Mean difference (MD) and standard mean difference (SMD) (IV, Random, 95% confidence interval (CI)). Test for overall effect was performed using Review Manager Software; (**D**): vitamin C increase; (**E**) MDA; (**F**) weight. Statistical method: SMD (IV, Random, 95% CI). Test for overall effect was performed using Review Manager Software.

The study by Khan (2014) [26] was divided in two parts (a, b) for analysis since it reports two concentrations of intervention juices (low blackcurrant and high blackcurrant juices, respectively) against placebo. For the trials that reported data on mean change from baseline for TEAC, SOD, or CAT (Figure 2A–C), no significant differences were found in the subjects who received juice supplements, as compared to control subjects, with standardized mean differences (SMD) effects (95% confidence interval (CI)), 0.17 (−0.26, 0.59), −1.49 (−25.69; 22.71), and 0.71 (−0.26; 1.67), respectively).

In addition, the mean weight change was reported in eight trials and was not significantly different between intervention and placebo (0.08 [−0.11, 0.26]). The effects of juice consumption on increasing levels of vitamin C and reduction of MDA were significantly different compared to those of placebo and in favor to intervention; the values of SMD (95% CI) were 1.46 (0.53, 2.39) and −1.48 (−2.39, −0.58), respectively (Figure 2D–F). These positive results came mostly from studies where the interventions were fruits juices (pomegranate [35,36], grapes [32], cranberries [12], and blackcurrants [26]). One study report data on tomato juice [21].

Some of the meta-analyses (increased vitamin C, MDA reduction, and CAT) presented I^2^ values higher than 50%, which represents a high heterogeneity between studies. The sensitivity analysis showed that more than one study was responsible for the increase in I^2^. This heterogeneity is acceptable and could be explained by the intrinsic characteristics of the included studies, conduction and design of trials, and type of intervention and outcomes analyzed.

Overall, our systematic review and meta-analysis showed that juices can have health benefits since their consumption significantly affected the plasma concentration of an antioxidant agent (vitamin C) and reduced MDA levels. Fruits, especially berries and pomegranate, showed greater association with these benefits.

**Table 1:** Characteristics of included studies.

Study (Ref.)	No. of Subjects	Country	Study Design	Population	Intervention	Control	Duration	Gender (M/F)	Age (Years) ± SD
**Amagase [11]**	50	China	Parallel	Healthy individuals	Goji berry juice 60 mL twice daily	Placebo juice	1 month	25 M: 25 F	58.1 ± 4.8
**Basu [12]**	36	USA	Parallel	Healthy women with metabolic syndrome	Cranberry juice 240 mL twice daily	Placebo juice	8 weeks	36 F	52.0 ± 8.0
**Brivida [13]**	22	Germany	Crossover	Healthy individuals	Tomato juice 330 mL daily	Carrot juice 330 mL/day	2 weeks	22 M	-
**Bub [14]**	27	Germany	Crossover	Healthy individuals	Juice A (mix of apple, mango, orange and blueberries)	Juice B (mix of apple, mango orange, lime and apricot)	2 weeks	27 M	35.0 ± 4.0
**Butalla [15]**	68	USA	Parallel	Overweight breast cancer survivors	Orange carrot juice	Purple carrot juice	3 weeks	68 F	56.5 ± 8.0
**Castilla [17]**	38	Spain	Parallel	Hemodialysis patients	Red grape juice 50 mL twice daily	Placebo juice	2 weeks	19 M: 19 F	60.6 ± 3.6
**Castilla [16]**	16	Spain	Parallel	Hemodialysis patients	Red grape juice 50 mL twice daily	Placebo juice	2 weeks	8 M: 8 F	33–79
**Duthie [18]**	20	UK	Parallel	Healthy individuals	Cranberry juice 250 mL three time daily	Placebo juice	2 weeks	20 F	27.8 ± 7.0
**Ellinger [19]**	12	Germany	Crossover	Healthy individuals	Juice blend (acai, camu-camu, blackberry) 400 mL daily	Placebo juice	1 day	6 M: 6 F	33.0 ± 7.0
**García-Alonso [20]**	18	Spain	Parallel	Healthy individuals	Tomato juice 300 mL daily	N-3 PUFA-enriched tomato juice 300 mL daily	2 weeks	18 F	35–55
**Ghavipour [21]**	64	Iran	Parallel	Healthy overweight or obese women	Tomato juice 330 mL daily	Placebo (water)	20 days	64 F	25.3 ± 0.7
**Guo [22]**	26	China	Parallel	Healthy elderly individuals	Pomegranate juice 250 mL daily	Apple juice 250 mL daily	4 weeks	20 M: 6 F	63.4 ± 4.5
**Guo [23]**	44	China	Crossover	Patients with fatty liver disease	Bayberry juice 250 mL twice daily	Placebo juice	2 weeks	12 M: 32 F	21.2 ± 1.2
**Jacob [24]**	24	Germany	Parallel	Healthy individuals	Tomato juice lycopene enriched 250 mL twice daily	Tomato juice vitamin C enriched 250 mL twice daily	2 weeks	4 M: 20 F	19–23
**Karlsen [25]**	63	Norway	Parallel	Healthy individuals with cardiovascular disease risk	Bilberry juice 330 mL daily	Placebo (water)	4 weeks	46 M: 17 F	50–70
**Khan [26]**	64	UK	Parallel	Healthy individuals	Blackcurrant juice (low and high concentration) 250 mL four times per day	Placebo juice	6 weeks	43M: 21 F	52.3 ± 9.7
**Kuntz [27]**	30	Germany	Crossover	Healthy individuals	Juice (red grapes and bilberries) 330 mL daily	Placebo juice	2 weeks	30 F	24.0 ± 1.2
**Lee [28]**	32	Korea	Parallel	Smokers	Carrot juice 300 mL daily	Placebo juice	8 weeks	-	35.6 ± 2.2
**Lynn [29]**	51	UK	Parallel	Healthy individuals	Pomegranate juice 330 mL daily	Placebo juice	4 weeks	16 M: 35 F	37.5 ± 1.1
**Mathison [30]**	12	USA	Crossover	Healthy individuals	Cranberry juice	Placebo juice	1 day	6M: 6F	27.5 ± 1.3
**Parashar [31]**	26	India	Parallel	Healthy elderly individuals	Pomegranate juice 250 mL daily	Orange juice 250 mL daily	4 weeks	20M: 6 F	63.5 ± 4.5
**Park [32]**	40	Korea	Parallel	Healthy individuals with borderline hypertension	Grape juice 400 mL daily	Placebo juice	8 weeks	40 M	44.5 ± 2.0
**Pourhamdi [33]**	80	Azerbaijan	Parallel	Healthy overweight or obese women	Tomato juice 330 mL daily	Placebo (water)	20 days	80 F	20–30
**Sabitha [34]**	33	India	Parallel	Diabetic mellitus type 2 patients	Noni juice 30 mL daily	Placebo juice	21 days	17 M: 16 F	-
**Shema-Didi [35]**	101	Israel	Parallel	Hemodialysis patients	Pomegranate juice 100 mL daily	Placebo juice	12 months	55 M: 46 F	66.7 ± 12.1
**Sohrab [36]**	44	Iran	Parallel	Diabetic mellitus type 2 patients	Pomegranate juice 250 mL daily	Placebo juice	12 weeks	23 M: 21 F	56.0 ± 6.8
**Soriano [37]**	20	Spain	Crossover	Healthy individuals	Apple juice, vitamin C enriched 250 mL twice daily	Apple juice polyphenol enriched 250 mL twice daily	2 weeks	8 M: 12 F	23.5 ± 2.1
**Uprichard [38]**	28	New Zealand	Parallel	Diabetic mellitus type 2 patients	Tomato juice 250 mL twice daily	Placebo juice	4 weeks	20 M: 8 F	61.5 ± 7.0

Although there are many studies in the literature on the preventive role of pomegranate in metabolic syndrome, obesity, hypertension, and cardiovascular disease, and other diseases, a cause and effect relationship between the consumption of fruit juice and the claimed health effects has not been established yet. It may be related to some antioxidant compounds, such as vitamin C [40,41,42,43,44]. In addition, grape and berry juices are rich sources of the antioxidant flavonoids catechin, epicatechin, quercetin, and anthocyanins. Some human studies showed promising antioxidant activity results with these fruits. However, limitations included the sample size, short duration of supplementation, and the fact that only few indexes of antioxidant status were measured in clinical trials [8,45].

In our meta-analysis, other antioxidant agents (SOD, CAT) and the TEAC levels were not influenced by beverage intake. A possibility is that fruit juice intervention might modestly increase the subject’s antioxidant capacity, which can also be influenced by diet, daily dosage of juice (which may or may not reflect phytochemical bioavailability), the participant’s baseline characteristics, and health conditions. The RCTs included in the analysis presented significant differences in sample size and population particularities (e.g., age, sex, and clinical conditions). Most studies had limited sample sizes of varied gender and ethnicity, in addition to short treatment periods and a focus on early biomarkers rather than functional endpoints. Moreover, the effects of the intervention on cellular and tissue levels may not appear immediately. Antioxidant effects *in vivo* are dependent on the bioavailability of antioxidants, as determined by uptake, distribution, metabolism, and excretion [10,45]. Therefore, the effects of juices in this study might be mildly underestimated.

Furthermore, our assessment of the effects of juices on other outcome measures related to oxidative stress and antioxidant capacity (e.g., FRAP (ferric reducing ability of plasma) assay, ORAC (oxygen radical absorbance capacity) assay, and LDL oxidation) was limited by the small number of studies currently available on this topic. Because of the difficulty in measuring each antioxidant component separately and interactions among antioxidants, these assays have been developed to measure the total antioxidant capacity of serum or plasma. Although some included studies reported data on these assays, there is a lack of standardization in proper reporting of data (e.g., measurement units) in the case of these methods in clinical trials and this can be a barrier to gathering evidence [8,40,46].

Concerning other effects of juices, it is known that drinks contribute approximately 25%–50% of water intake; thus, they are important for meeting daily water needs and achieving hydration [47]. However, it has been argued that drinks contribute significantly to the increasing obesity rates worldwide, mainly because of their energy content and additionally supplemented sugars [47]. Weight gain and obesity are conditions related to oxidative stress. Concerning the meta-analysis of weight change, no differences were observed between interventions and placebo for any included study. Therefore, the daily consumption of juices, while avoiding excess consumption, is recommended.

The accumulating evidence for the involvement of oxidative stress in the pathogenesis of various diseases is an expanding field for scientists and health professionals. The studies examining the effect of juice consumption are varied in design and methodology. Natural antioxidants from fruits and vegetables present beneficial effects supported mostly by epidemiological studies [10,44]. Hence, they should be investigated further by well-designed, large-scale double-blind, randomized, controlled clinical trials and in other meta-analyses. In addition, extension studies should be performed in order to demonstrate the effects of interventional juices over time and in safety parameters (such as related to adverse events, allergies or problems of juice consumption) for participants.

## 3. Experimental Section

To conduct the systematic review, searches were performed in PubMed, Scopus, International Pharmaceutical Abstracts and SciELO, along with manual searching, in July 2015 with no time limits. The following search terms were used: clinical, trial, random*, antioxidant*, juice, beverage in combination with the Boolean operators AND and OR. We included RCTs conducted in human subjects, comparing the consumption of any fruit or vegetable juice with placebo, water or other controlled drink. Articles published in non-Roman characters were excluded. Following PRISMA (Preferred Reporting Items for Systematic Reviews and Meta-Analyses) recommendations, [48] titles and abstracts of records retrieved were initially screened and the full text of those considered relevant were then analyzed. The literature selection process was conducted by two independent reviewers, with a third reviewer in case of discrepancies.

### 3.1. Data Extraction and Quality Assessment

Data on the mean change of levels of SOD and CAT enzymes, as well as vitamin C, were collected as the outcome measures. These substances are antioxidant agents that protect cells and tissues from free radical damage. Another outcome measure extracted from the articles was the results on MDA changes. This substance is an oxidant marker of lipid peroxidation and its reduction in plasma is beneficial. Data from the TEAC, as a method to assess the global antioxidant plasma capacity, were also extracted [44,46].

Mean change from baseline in the weight of subjects was another main outcome since overweight and obesity can be related to chronic diseases and oxidative stress [10,43]. Two researchers performed data extraction independently, with a third reviewer resolving any discrepancies. The quality of the included studies was evaluated using two different tools: The Jadad Scale [39] and the Cochrane Collaboration’s tool for assessing the risk of bias [49]. The results of the quality assessment were used to derive conclusions accordingly, but no studies were excluded.

### 3.2. Data Analysis

When possible, pairwise meta-analysis of the included RCTs using a placebo as comparator were performed for the main outcome measures. For each meta-analysis, the primary effect size was the MD or SMD and its 95% CI. Pool effect size was calculated using the inverse variance method (fixed or random effects model) and *p* < 0.05 (two-tailed) was considered statistically significant. Between-trial heterogeneity was estimated using the I^2^ measure (I^2^ > 50%, significant heterogeneity) [50]. To evaluate the impact of any study on data heterogeneity, sensitivity analyses consisting of the hypothetical sequential removal of one study at a time were done. Statistical analysis was performed using Review Manager version 5.1 (The Nordic Cochrane Centre, The Cochrane Collaboration, Copenhagen, Denmark).

## 4. Conclusions

Collectively, the data presented in this systematic review and meta-analysis suggest that some potential health-related and disease prevention markers associated with consumption of juices should be further investigated.

Some types of fruit and vegetable juices could increase the antioxidant capacity in the plasma of healthy individuals and patients with chronic conditions, as evidenced by the increase of vitamin C levels and decrease in MDA. These data showed a potential function of juices as antioxidant beverages. Further studies are still needed to clarify the molecular processes and signaling pathways involved in the antioxidant capacity of fruit and vegetables juices in the human body.

Despite this potential, there are many unanswered questions related to fruit and vegetables juices and health in humans. There is a clear need for larger, well-controlled studies of longer duration with well-defined outcomes. Furthermore, it is important to determine an appropriate dose of juices that reflects bioavailability of phytochemicals and antioxidant agents in human plasma. The implications of the baseline health status should also be considered, since healthy individuals and patients with chronic conditions may not have the same antioxidant status.

## Supplementary Materials

Supplementary materials can be accessed at: http://www.mdpi.com/1420-3049/20/12/19834/s1.

## References

[1] Lobo V., Patil A., Phatak A., Chandra N. (2010). Free radicals, antioxidants and functional foods: Impact on human health. Pharmacogn. Rev..

[2] White P.A.S., Oliveira R.M.C., Oliveira A.P., Serafini M.R., Araújo A.A.S., Gelain D.P., Moreira J.C.F., Almeida J.R.G.S. (2014). Antioxidant activity and mechanisms of action of natural compounds isolated from lichens: A systematic review. Molecules.

[3] Bhardwaj R.L., Pandey S. (2011). Juice blends—A way of utilization of under-utilized fruits, vegetables, and spices: A review. Crit. Rev. Food Sci. Nutr..

[4] Singh G.M., Micha R., Khatibzadeh S., Shi P., Lim S., Andrews K.G., Engell R.E., Ezzati M., Mozaffarian D. (2015). Global, regional, and national consumption of sugar-sweetened beverages, fruit juices, and milk: A systematic assessment of beverage intake in 187 countries. PLoS ONE.

[5] Hyson D.A. (2011). A comprehensive review of apples and apple components and their relationship to human health. Adv. Nutr..

[6] Dumbravă D.G., Hădărugă N.G., Moldovan C., Raba D.M., Popa M.V., Rădoi B. (2011). Antioxidant activity of some fresh vegetables and fruits juices. J. Agroaliment. Process. Technol..

[7] Slavin J.L., Lloyd B. (2012). Health benefits of fruits and vegetables. Adv. Nutr..

[8] Hyson D.A. (2015). A review and critical analysis of the scientific literature related to 100% fruit juice and human health. Adv. Nutr..

[9] Wang Y.C., Bleich S.N., Gortmaker S.L. (2008). Increasing caloric contribution from sugar-sweetened beverages and 100% fruit juices among us children and adolescents 1988–2004. Pediatrics.

[10] Morita M., Naito Y., Yoshikawab T., Niki E. (2015). Assessment of radical scavenging capacity of antioxidants contained in foods and beverages in plasma solution. Food Funct..

[11] Amagase H., Sun B., Borek C. (2009). Lycium barbarum (goji) juice improves *in vivo* antioxidant biomarkers in serum of healthy adults. Nutr. Res..

[12] Basu A., Betts N.M., Ortiz J., Simmons B., Wu M., Lyons T.J. (2011). Low-energy cranberry juice decreases lipid oxidation and increases plasma antioxidant capacity in women with metabolic syndrome. Nutr. Res..

[13] Briviba K., Schnäbele K., Rechkemmer G., Bub A. (2004). Supplementation of a diet low in carotenoids with tomato or carrot juice does not affect lipid peroxidation in plasma and feces of healthy men. J. Nutr..

[14] Bub A., Watzl B., Blockhaus M., Briviba K., Liegibel U., Müller H., Pool-Zobel B.L., Rechkemmer G. (2003). Fruit juice consumption modulates antioxidative status, immune status and DNA damage. J. Nutr. Biochem..

[15] Butalla A.C., Crane T.E., Patil B., Wertheim B.C., Thompson P., Thomson C.A. (2012). Effects of a carrot juice intervention on plasma carotenoids, oxidative stress, and inflammation in overweight breast cancer survivors. Nutr. Cancer.

[16] Castilla P., Dávalos A., Teruel J.L., Cerrato F., Fernández-Lucas M., Merino J.L., Sánchez-Martín C.C., Ortuño J., Lasunción M.A. (2008). Comparative effects of dietary supplementation with red grape juice and vitamin e on production of superoxide by circulating neutrophil nadph oxidase in hemodialysis patients. Am. J. Clin. Nutr..

[17] Castilla P., Echarri R., Dávalos A., Cerrato F., Ortega H., Teruel J.L., Lucas M.F., Gómez-Coronado D., Ortuño J., Lasunción M.A. (2006). Concentrated red grape juice exerts antioxidant, hypolipidemic, and antiinflammatory effects in both hemodialysis patients and healthy subjects. Am. J. Clin. Nutr..

[18] Duthie S.J., Jenkinson A.M., Crozier A., Mullen W., Pirie L., Kyle J., Yap L.S., Christen P., Duthie G.G. (2006). The effects of cranberry juice consumption on antioxidant status and biomarkers relating to heart disease and cancer in healthy human volunteers. Eur. J. Nutr..

[19] Ellinger S., Gordon A., Kürten M., Jungfer E., Zimmermann B.F., Zur B., Ellinger J., Marx F., Stehle P. (2012). Bolus consumption of a specifically designed fruit juice rich in anthocyanins and ascorbic acid did not influence markers of antioxidative defense in healthy humans. J. Agric. Food Chem..

[20] García-Alonso F.J., Jorge-Vidal V., Ros G., Periago M.J. (2012). Effect of consumption of tomato juice enriched with n-3 polyunsaturated fatty acids on the lipid profile, antioxidant biomarker status, and cardiovascular disease risk in healthy women. Eur. J. Nutr..

[21] Ghavipour M., Sotoudeh G., Ghorbani M. (2015). Tomato juice consumption improves blood antioxidative biomarkers in overweight and obese females. Clin. Nutr..

[22] Guo C., Wei J., Yang J., Xu J., Pang W., Jiang Y. (2008). Pomegranate juice is potentially better than apple juice in improving antioxidant function in elderly subjects. Nutr. Res..

[23] Guo H., Zhong R., Liu Y., Jiang X., Tang X., Li Z., Xia M., Ling W. (2014). Effects of bayberry juice on inflammatory and apoptotic markers in young adults with features of non-alcoholic fatty liver disease. Nutrition.

[24] Jacob K., Periago M.J., Böhm V., Berruezo G.R. (2008). Influence of lycopene and vitamin c from tomato juice on biomarkers of oxidative stress and inflammation. Br. J. Nutr..

[25] Karlsen A., Paur I., Bøhn S.K., Sakhi A.K., Borge G.I., Serafini M., Erlund I., Laake P., Tonstad S., Blomhoff R. (2010). Bilberry juice modulates plasma concentration of NF-κB related inflammatory markers in subjects at increased risk of cvd. Eur. J. Nutr..

[26] Khan F., Ray S., Craigie A.M., Kennedy G., Hill A., Barton K.L., Broughton J., Belch J.J.F. (2014). Lowering of oxidative stress improves endothelial function in healthy subjects with habitually low intake of fruit and vegetables: A randomized controlled trial of antioxidant- and polyphenol-rich blackcurrant juice. Free Radic. Biol. Med..

[27] Kuntz S., Kunz C., Herrmann J., Borsch C.H., Abel G., Fröhling B., Dietrich H., Rudloff S. (2014). Anthocyanins from fruit juices improve the antioxidant status of healthy young female volunteers without affecting anti-inflammatory parameters: Results from the randomised, double-blind, placebo-controlled, cross-over anthonia (anthocyanins in nutrition investigation alliance) study. Br. J. Nutr..

[28] Lee H.J., Park Y.K., Kang M.H. (2011). The effect of carrot juice, beta-carotene supplementation on lymphocyte DNA damage, erythrocyte antioxidant enzymes and plasma lipid profiles in korean smoker. Nutr. Res. Pract..

[29] Lynn A., Hamadeh H., Leung W.C., Russell J.M., Barker M.E. (2012). Effects of pomegranate juice supplementation on pulse wave velocity and blood pressure in healthy young and middle-aged men and women. Plant Foods Hum. Nutr..

[30] Mathison B.D., Kimble L.L., Kaspar K.L., Khoo C., Chew B.P. (2014). Consumption of cranberry beverage improved endogenous antioxidant status and protected against bacteria adhesion in healthy humans: A randomized controlled trial. Nutr. Res..

[31] Parashar A., Badal S. (2011). Pomegranate juice is potentially better than orange juice in improving antioxidant function in elderly subjects. Pharm. Res..

[32] Park Y.K., Lee S.H., Park E., Kim J.S., Kang M.H. (2009). Changes in antioxidant status, blood pressure, and lymphocyte DNA damage from grape juice supplementation. Ann. N. Y. Acad. Sci..

[33] Pourahmadi Z., Mahboob S., Saedisomeolia A., Reykandeh M.T. (2015). The effect of tomato juice consumption on antioxidant status in overweight and obese females. Women Health.

[34] Sabitha P., Adhikari Prabha M.R., Shetty Rukmini M.S., Anupama H., Asha K. (2009). The beneficial effects of noni fruit juice in diabetic patients. J. Clin. Diagn. Res..

[35] Shema-Didi L., Sela S., Ore L., Shapiro G., Geron R., Moshe G., Kristal B. (2012). One year of pomegranate juice intake decreases oxidative stress, inflammation, and incidence of infections in hemodialysis patients: A randomized placebo-controlled trial. Free Radic. Biol. Med..

[36] Sohrab G., Angoorani P., Tohidi M., Tabibi H., Kimiagar M., Nasrollahzadeh J. (2015). Pomegranate (punicagranatum) juice decreases lipid peroxidation, but has no effect on plasma advanced glycated end-products in adults with type 2 diabetes: A randomized double-blind clinical trial. Food Nutr. Res..

[37] Soriano-Maldonado A., Hidalgo M., Arteaga P., de Pascual-Teresa S., Nova E. (2014). Effects of regular consumption of vitamin c-rich or polyphenol-rich apple juice on cardiometabolic markers in healthy adults: A randomized crossover trial. Eur. J. Nutr..

[38] Upritchard J.E., Sutherland W.H.F., Mann J.I. (2000). Effect of supplementation with tomato juice, vitamin e, and vitamin c on ldl oxidation and products of inflammatory activity in type 2 diabetes. Diabetes Care.

[39] Jadad A., Moore R., Carroll D., Jenskinson C., Reynold D., Gavaghan D. (1996). Assessing the quality of reports of randomized clinical trials: Is blinding necessary?. Control Clin. Trials.

[40] Liu R.H. (2013). Dietary bioactive compounds and their health implications. J. Food Sci..

[41] Wang B., Liu K., Mi M., Wang J. (2014). Effect of fruit juice on glucose control and insulin sensitivity in adults: A meta-analysis of 12 randomized controlled trials. PLoS ONE.

[42] Danesi F., Kroon P.A., Saha S., de Biase D., D’Antuono L.F., Bordoni A. (2014). Mixed pro- and anti-oxidative effects of pomegranate polyphenols in cultured cells. Int. J. Mol. Sci..

[43] Moreno L.A., Gottrand F., Huybrechts I., Ruiz J.R., González-Gross M. (2014). Nutrition and lifestyle in european adolescents: The helena (healthy lifestyle in Europe by nutrition in adolescence) study. Adv. Nutr..

[44] Araújo K.L.G.V., Magnani M.M., Nascimento J.A., Souza A.L., Epaminondas P.S., Souza A.L., Queiroz N., Souza A.G. (2014). Antioxidant activity of co-products from guava, mango and barbados cherry produced in the brazilian northeas. Molecules.

[45] O’Byrne D.J., Devaraj S., Grundy S.M., Jialal I. (2002). Comparison of the antioxidant effects of concord grape juice flavonoids and a-tocopherol on markers of oxidative stress in healthy adults. Am. J. Clin. Nutr..

[46] Huang D., Ou B., Prior R.L. (2005). The chemistry behind antioxidant capacity assays. J. Agric. Food Chem..

[47] Malisova O., Bountziouka B., Zampelas A., Kapsokefalou M. (2015). Evaluation of drinks contribution to energy intake in summer and winter. Nutrients.

[48] Liberati A., Altman D., Tetzlaff J., Mulrow C., Gotzsche P., Ioannidis J. (2009). Prisma statement for reporting systematic reviews and meta-analyses of studies that evaluate health care interventions: Explanation and elaboration. Ann. Intern. Med..

[49] Higgins J., Green S. (2011). Cochrane Handbook for Systematic Reviews of Interventions Version 5.1.0.

[50] Huedo-Medina T., Sánchez-Meca J., Marín-Martínez F., Botella J. (2006). Assessing heterogeneity in meta-analysis: Q statistic or i2 index?. Psychol. Methods.

