# Supplementary material for: Impact of Natural Juice Consumption on Plasma Antioxidant Status: A Systematic Review and Meta-Analysis
Source: Molecules. 2015 Dec 10;20(12):22146–56. doi: 10.3390/molecules201219834 (PMC6331866; doi:10.3390/molecules201219834)
Supplement: Supplementary file 1 [file molecules-20-19834-s001.pdf]

Supplementary Materials: Impact of Natural Juice Consumption on Plasma Antioxidant Status: A Systematic Review and Meta-Analysis

Fernanda S. Tonin, Laiza M. Steimbach, Astrid Wiens, Cássio M. Perlin and Roberto Pontarolo

Table S1. Main reasons for study exclusion.

**Reason for Exclusion**	**Number of Studies**
Type of study or study design (e.g., reviews, trials without a control group or not randomized)	21
Type of intervention different from fruit or vegetables juice (e.g., capsules, plant extracts)	12
Outcome measures not related to oxidative stress or antioxidant capacity in human plasma	11
Impossibility of data collection	4
Population (e.g., athletes)	3

Table S2. Jadad Scale. Scores obtained for each study included in the systematic review.

STUDY [Ref]	1. Was the Study Described as Randomized?	2. The Randomization Method was Described in the Paper, and that Method was Appropriate.	3. Was the Study Described as Double Blind?	4. The Method of Blinding was Described, and It was Appropriate.	5. Was there a Description of Withdrawals and Dropouts?	SCORE
**Amagase 2009**	YES	NOT MENTIONED	YES	NOT MENTIONED	YES	3
**Brivida 2004**	YES	NOT MENTIONED	NOT MENTIONED	NOT MENTIONED	NOT MENTIONED	1
**Bub 2003**	YES	NOT MENTIONED	NOT MENTIONED	NOT MENTIONED	NOT MENTIONED	1
**Duthie 2006**	YES	NOT MENTIONED	YES	NOT MENTIONED	NO	2
**Ellinger 2012**	YES	YES	NOT MENTIONED	NOT MENTIONED	NOT MENTIONED	2
**García-A. 2012**	YES	NOT MENTIONED	NO	NOT MENTIONED	YES	2
**Ghavipour 2014**	YES	NOT MENTIONED	NO	NOT MENTIONED	YES	2
**Guo 2008**	YES	NOT MENTIONED	NO	NOT MENTIONED	NO	1
**Jacob 2008**	YES	NOT MENTIONED	NO	NOT MENTIONED	NO	1
**Khan 2014**	YES	YES	YES	NOT MENTIONED	YES	4
**Kuntz 2014**	YES	YES	YES	NOT MENTIONED	NOT MENTIONED	3
**Lynn 2012**	YES	YES	NO	NOT MENTIONED	YES	3
**Mathison 2014**	YES	YES	YES	NOT MENTIONED	NOT MENTIONED	3
**Parashar 2011**	YES	NOT MENTIONED	NO	NOT MENTIONED	NO	1
**Park 2009**	YES	NOT MENTIONED	YES	NOT MENTIONED	NO	2
**Pourhamdi, 2015**	YES	YES	YES	NOT MENTIONED	YES	4
**Soriano-M. 2014**	YES	NOT MENTIONED	NOT MENTIONED	NOT MENTIONED	YES	2
**Butalla 2012**	YES	NOT MENTIONED	NO	NOT MENTIONED	YES	2
**Basu 2011**	YES	NOT MENTIONED	NOT MENTIONED	NOT MENTIONED	YES	2
**Castilla 2006**	YES	NOT MENTIONED	NO	NOT MENTIONED	YES	2
**Castilla 2008**	YES	NOT MENTIONED	NO	NOT MENTIONED	YES	2
**Guo 2014**	YES	NOT MENTIONED	YES	NOT MENTIONED	YES	3
**Karlsen 2010**	YES	NOT MENTIONED	NOT MENTIONED	NOT MENTIONED	YES	2
**Lee 2011**	YES	NOT MENTIONED	NOT MENTIONED	NOT MENTIONED	NOT MENTIONED	1
**Sabitha 2009**	YES	NOT MENTIONED	YES	NOT MENTIONED	NOT MENTIONED	2
**Shema-Didi 2012**	YES	YES	YES	NOT MENTIONED	YES	4
**Sohrab 2015**	YES	NOT MENTIONED	YES	NOT MENTIONED	YES	3
**Uprichart 2000**	YES	NOT MENTIONED	NO	NOT MENTIONED	YES	2

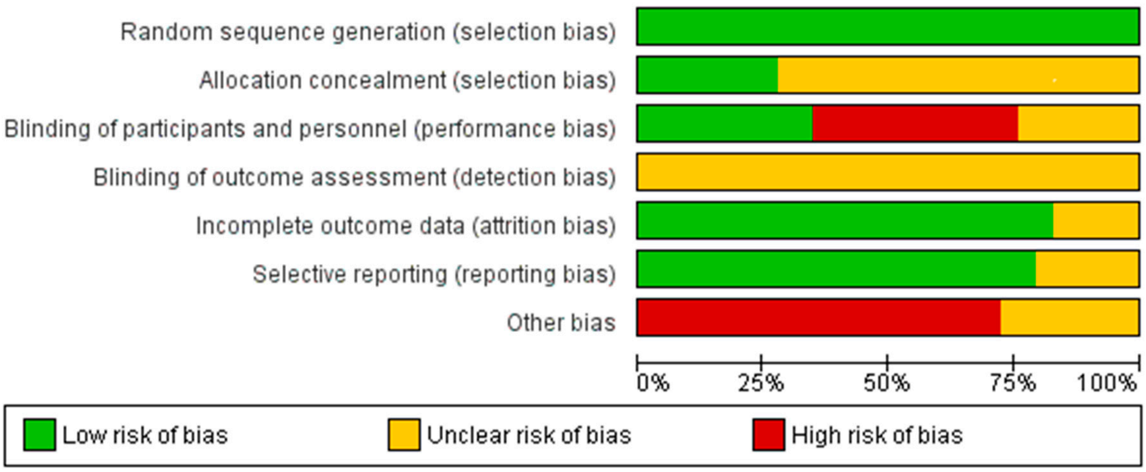

Figure S1 Risk of bias graph of all included studies according to the Cochrane Collaboration.

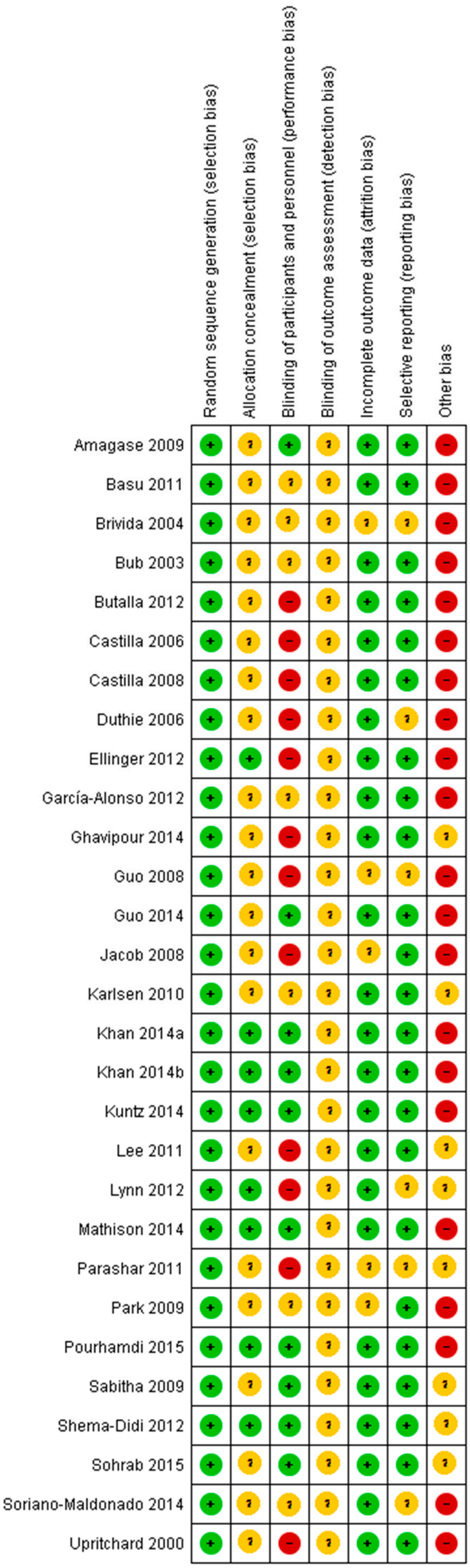

Figure S2. Risk of bias summary of all included studies.

Appendix 1—List of Excluded Studies [1–51]

1. Abbey, M.; Noakes, M.; Nestel, P.J. Dietary supplementation with orange and carrot juice in cigarette smokers lowers oxidation products in copper-oxidized low-density lipoproteins. *J. Am. Diet. Assoc.* **1995**, *95*, 671–675.
2. Agarwal, S.; Rao, A.V. Tomato lycopene and low density lipoprotein oxidation: A human dietary intervention study. *Lipids* **1998**, *33*, 981–984.
3. Aiso, I.; Inoue, H.; Seiyama, Y.; Kuwano, T. Compared with the intake of commercial vegetable juice, the intake of fresh fruit and komatsuna (brassica rapa l. Var. Perviridis) juice mixture reduces serum cholesterol in middle-aged men: A randomized controlled pilot study. *Lipids Health Dis.* **2014**, *13*, doi:10.1186/1476-511X-13-102.
4. Alvarez-Parrilla, E.; De La Rosa, L.A.; Legarreta, P.; Saenz, L.; Rodrigo-García, J.; Gonzlez-Aguilar, G.A. Daily consumption of apple, pear and orange juice differently affects plasma lipids and antioxidant capacity of smoking and non-smoking adults. *Int. J. Food Sci. Nutr.* **2010**, *61*, 369–380.
5. Aptekmann, N.P.; Cesar, T.B. Orange juice improved lipid profile and blood lactate of overweight middle-aged women subjected to aerobic training. *Maturitas* **2010**, *67*, 343–347.
6. Arendt, B.M.; Boetzer, A.M.; Lemoch, H.; Winkler, P.; Rockstroh, J.K.; Berthold, H.K.; Spengler, U.; Goerlich, R. Plasma antioxidant capacity of hiv-seropositive and healthy subjects during long-term ingestion of fruit juices or a fruit-vegetable-concentrate containing antioxidant polyphenols. *Eur. J. Clin. Nutr.* **2001**, *55*, 786–792.
7. Bamonti, F.; Novembrino, C.; Ippolito, S.; Soresi, E.; Ciani, A.; Lonati, S.; Scurati-Manzoni, E.; Cighetti, G. Increased free malondialdehyde concentrations in smokers normalise with a mixed fruit and vegetable juice concentrate: A pilot study. *Clin. Chem. Lab. Med.* **2006**, *44*, 391–395.
8. Böhm, V.; Bitsch, R. Intestinal absorption of lycopene from different matrices and interactions to other carotenoids, the lipid status, and the antioxidant capacity of human plasma. *Eur. J. Nutr.* **1999**, *38*, 118–125.
9. Bub, A.; Watzl, B.; Abrahamse, L.; Delincee, H.; Adam, S.; Wever, J.; Muller, H.; Rechkemmer, G. Moderate intervention with carotenoid-rich vegetable products reduces lipid peroxidation in men. *J. Nutr.* **2000**, *130*, 2200–2206.
10. Collins, J.K.; Arjmandi, B.H.; Claypool, P.L.; Perkins-Veazie, P.; Baker, R.A.; Clevidence, B.A. Lycopene from two food sources does not affect antioxidant or cholesterol status of middle-aged adults. *Nutr. J.* **2004**, *3*, doi:10.1186/1475-2891-3-15.
11. Dalgård, C.; Nielsen, F.; Morrow, J.D.; Enghusen-Poulsen, H.; Jonung, T.; Hørder, M.; De Maat, M.P.M. Supplementation with orange and blackcurrant juice, but not vitamin e, improves inflammatory markers in patients with peripheral arterial disease. *Br. J. Nutr.* **2009**, *101*, 263–269.
12. Diaz-Rubio, M.E.; Perez-Jimenez, J.; Martinez-Bartolome, M.A.; Alvarez, I.; Saura-Calixto, F. Regular consumption of an antioxidant-rich juice improves oxidative status and causes metabolome changes in healthy adults. *Plant Foods Hum. Nutr.* **2015**, *70*, 9–14.
13. Eccleston, C.; Baoru, Y.; Tahvonen, R.; Kallio, H.; Rimbach, G.H.; Minihane, A.M. Effects of an antioxidant-rich juice (sea buckthorn) on risk factors for coronary heart disease in humans. *J. Nutr. Biochem.* **2002**, *13*, 346–354.
14. George, T.W.; Waroonphan, S.; Niwat, C.; Gordon, M.H.; Lovegrove, J.A. Effects of acute consumption of a fruit and vegetable puree-based drink on vasodilation and oxidative status. *Br. J. Nutr.* **2013**, *109*, 1442–1452.
15. Ghanim, H.; Sia, C.L.; Upadhyay, M.; Korzeniewski, K.; Viswanathan, P.; Abuaysheh, S.; Mohanty, P.; Dandona, P. Orange juice neutralizes the proinflammatory effect of a high-fat, high-carbohydrate meal and prevents endotoxin increase and toll-like receptor expression. *Am. J. Clin. Nutr.* **2010**, *91*, 940–949.
16. Gorinstein, S.; Caspi, A.; Libman, I.; Katrich, E.; Lerner, H.T.; Trakhtenberg, S. Fresh israeli jaffa sweetie juice consumption improves lipid metabolism and increases antioxidant capacity in hypercholesterolemic patients suffering from coronary artery disease: Studies *in vitro* and in humans and positive changes in albumin and fibrinogen fractions. *J. Agric. Food Chem.* **2004**, *52*, 5215–5222.
17. Heber, D.; Seeram, N.P.; Wyatt, H.; Henning, S.M.; Zhang, Y.; Ogden, L.G.; Dreher, M.; Hill, J.O. Safety and antioxidant activity of a pomegranate ellagitannin-enriched polyphenol dietary supplement in overweight individuals with increased waist size. *J. Agric. Food Chem.* **2007**, *55*, 10050–10054.
18. Hyson, D.; Studebaker-Hallman, D.; Davis, P.A.; Gershwin, M.E. Apple juice consumption reduces plasma low-density lipoprotein oxidation in healthy men and women. *J. Med. Food* **2000**, *3*, 159–166.

19. Inoue, T.; Komoda, H.; Uchida, T.; Node, K. Tropical fruit camu-camu (myrciaria dubia) has anti-oxidative and anti-inflammatory properties. *J. Cardiol.* **2008**, *52*, 127–132.
20. Jensen, G.S.; Wu, X.; Patterson, K.M.; Barnes, J.; Carter, S.G.; Scherwitz, L.; Beaman, R.; Endres, J.R.; Schauss, A.G. *In vitro* and *in vivo* antioxidant and anti-inflammatory capacities of an antioxidant-rich fruit and berry juice blend. Results of a pilot and randomized, double-blinded, placebo-controlled, crossover study. *J. Agric. Food Chem.* **2008**, *56*, 8326–8333.
21. Jeon, G.I.; Shin, M.J.; Lee, K.H.; Park, E. Effect of onion juice supplementation on antioxidant status in participants with mild hypercholesterolemia. *Food Sci. Biotechnol.* **2013**, *22*, 227–231.
22. Johnston, C.S.; Dancho, C.L.; Strong, G.M. Orange juice ingestion and supplemental vitamin c are equally effective at reducing plasma lipid peroxidation in healthy adult women. *J. Am. Coll. Nutr.* **2003**, *22*, 519–523.
23. Kardum, N.; Konić-Ristić, A.; Šavikin, K.; Spasić, S.; Stefanović, A.; Ivanišević, J.; Miljković, M. Effects of polyphenol-rich chokeberry juice on antioxidant/pro-oxidant status in healthy subjects. *J. Med. Food* **2014**, *17*, 869–874.
24. Kiefer, I.; Prock, P.; Lawrence, C.; Wise, J.; Bieger, W.; Bayer, P.; Rathmanner, T.; Kunze, M.; Rieder, A. Supplementation with mixed fruit and vegetable juice concentrates increased serum antioxidants and folate in healthy adults. *J. Am. Coll. Nutr.* **2004**, *23*, 205–211.
25. Knab, A.M.; Nieman, D.C.; Gillitt, N.D.; Shanely, R.A.; Cialdella-Kam, L.; Henson, D.A.; Sha, W. Effects of a flavonoid-rich juice on inflammation, oxidative stress, and immunity in elite swimmers: A metabolomics-based approach. *Int. J. Sport Nutr. Exerc. Metab.* **2013**, *23*, 150–160.
26. Lynn, A.; Mathew, S.; Moore, C.T.; Russell, J.; Robinson, E.; Soumpasi, V.; Barker, M.E. Effect of a tart cherry juice supplement on arterial stiffness and inflammation in healthy adults: A randomised controlled trial. *Plant Foods Hum. Nutr.* **2014**, *69*, 122–127.
27. Mackinnon, E.S.; Rao, A.V.; Josse, R.G.; Rao, L.G. Supplementation with the antioxidant lycopene significantly decreases oxidative stress parameters and the bone resorption marker n-telopeptide of type i collagen in postmenopausal women. *Osteoporos. Int.* **2011**, *22*, 1091–1101.
28. Maruyama, C.; Imamura, K.; Oshima, S.; Suzukawa, M.; Egami, S.; Tonomoto, M.; Baba, N.; Harada, M.; Ayaori, M.; Inakuma, T.; *et al.* Effects of tomato juice consumption on plasma and lipoprotein carotenoid concentrations and the susceptibility of low density lipoprotein to oxidative modification. *J. Nutr. Sci. Vitaminol. (Tokyo)* **2001**, *47*, 213–221.
29. Miglio, C.; Peluso, I.; Raguzzini, A.; Villaño, D.V.; Cesqui, E.; Catasta, G.; Toti, E.; Serafini, M. Fruit juice drinks prevent endogenous antioxidant response to high-fat meal ingestion. *Br. J. Nutr.* **2014**, *111*, 294–300.
30. Müller, L.; Theile, K.; Finze, S.; Böhm, V. Antioxidant capacity and antioxidant vitamins in human plasma as affected by intervention with a multicomponent beverage rich in vitamin c and vitamin e. *Ernahrung* **2011**, *35*, 101–110.
31. Murkovic, M.; Abuja, P.M.; Bergmann, A.R.; Zirngast, A.; Adam, U.; Winklhofer-Roob, B.M.; Toplak, H. Effects of elderberry juice on fasting and postprandial serum lipids and low-density lipoprotein oxidation in healthy volunteers: A randomized, double-blind, placebo-controlled study. *Eur. J. Clin. Nutr.* **2004**, *58*, 244–249.
32. O'Byrne, D.J.; Devaraj, S.; Grundy, S.M.; Jialal, I. Comparison of the antioxidant effects of concord grape juice flavonoids alpha-tocopherol on markers of oxidative stress in healthy adults. *Am. J. Clin. Nutr.* **2002**, *76*, 1367–1374.
33. Pedersen, C.B.; Kyle, J.; Jenkinson, A.M.; Gardner, P.T.; McPhail, D.B.; Duthie, G.G. Effects of blueberry and cranberry juice consumption on the plasma antioxidant capacity of healthy female volunteers. *Eur. J. Clin. Nutr.* **2000**, *54*, 405–408.
34. Peluso, I.; Villano, D.V.; Roberts, S.A.; Cesqui, E.; Raguzzini, A.; Borges, G.; Crozier, A.; Catasta, G.; Toti, E.; Serafini, M. Consumption of mixed fruit-juice drink and vitamin c reduces postprandial stress induced by a high fat meal in healthy overweight subjects. *Curr. Pharm. Des.* **2014**, *20*, 1020–1024.
35. Pilaczynska-Szczesniak, L.; Skarpanska-Steinborn, A.; Deskur, E.; Basta, P.; Horoszkiewicz-Hassan, M. The influence of chokeberry juice supplementation on the reduction of oxidative stress resulting from an incremental rowing ergometer exercise. *Int. J. Sport Nutr. Exerc. Metab.* **2005**, *15*, 48–58.
36. Pittaluga, M.; Sgadari, A.; Tavazzi, B.; Fantini, C.; Sabatini, S.; Ceci, R.; Amorini, A.M.; Parisi, P.; Caporossi, D. Exercise-induced oxidative stress in elderly subjects: The effect of red orange supplementation on the biochemical and cellular response to a single bout of intense physical activity. *Free Radic. Res.* **2013**, *47*, 202–211.

37. Riso, P.; Visioli, F.; Gardana, C.; Grande, S.; Brusamolino, A.; Galvano, F.; Galvano, G.; Porrini, M. Effects of blood orange juice intake on antioxidant bioavailability and on different markers related to oxidative stress. *J. Agric. Food Chem.* **2005**, *53*, 941–947.
38. Rosenblat, M.; Hayek, T.; Aviram, M. Anti-oxidative effects of pomegranate juice (pj) consumption by diabetic patients on serum and on macrophages. *Atherosclerosis* **2006**, *187*, 363–371.
39. Ruel, G.; Lapointe, A.; Pomerleau, S.; Couture, P.; Lemieux, S.; Lamarche, B.; Couillard, C. Evidence that cranberry juice may improve augmentation index in overweight men. *Nutr. Res.* **2013**, *33*, 41–49.
40. Samman, S.; Sivarajah, G.; Man, J.C.; Ahmad, Z.I.; Petocz, P.; Caterson, I.D. A mixed fruit and vegetable concentrate increases plasma antioxidant vitamins and folate and lowers plasma homocysteine in men. *J. Nutr.* **2003**, *133*, 2188–2193.
41. Schauss, A.G.; Jensen, G.S.; Wu, X. Increased antioxidant capacity and inhibition of lipid peroxidation in healthy adults consuming an açai (euterpe oleracea) fruit-based juice. *Acta Hortic.* **2009**, *841*, 97–100.
42. Shidfar, F. The effects of tomato consumption on serum glucose, apolipoprotein b, apolipoprotein a-i, homocysteine and blood pressure in type 2 diabetic patients. *Int. J. Food Sci. Nutr.* **2011**, *62*, 289–294.
43. Shidfar, F. The effects of cranberry juice on serum glucose, apob, apoa-i, lp(a), and paraoxonase-1 activity in type 2 diabetic male patients. *J. Res. Med. Sci.* **2012**, *17*, 355–360.
44. Silaste, M.L.; Alfthan, G.; Aro, A.; Kesaniemi, Y.A.; Horkko, S. Tomato juice decreases ldl cholesterol levels and increases ldl resistance to oxidation. *Br. J. Nutr.* **2007**, *98*, 1251–1258.
45. Snyder, S.M.; Reber, J.D.; Freeman, B.L.; Orgad, K.; Eggett, D.L.; Parker, T.L. Controlling for sugar and ascorbic acid, a mixture of flavonoids matching navel oranges significantly increases human postprandial serum antioxidant capacity. *Nutr. Res.* **2011**, *31*, 519–526.
46. Traustadottir, T.; Davies, S.S.; Stock, A.A.; Su, Y.; Heward, C.B.; Roberts, L.J., 2nd; Harman, S.M. Tart cherry juice decreases oxidative stress in healthy older men and women. *J. Nutr.* **2009**, *139*, 1896–1900.
47. Valentová, K.; Stejskal, D.; Bednář, P.; Vostálová, J.; Číhalík, Č.; Večeřová, R.; Koukalová, D.; Kolář, M.; Reichenbach, R.; Škňouřil, L.; *et al.* Biosafety, antioxidant status, and metabolites in urine after consumption of dried cranberry juice in healthy women: A pilot double-blind placebo-controlled trial. *J. Agric. Food Chem.* **2007**, *55*, 3217–3224.
48. Vieira, F.G.; Di Pietro, P.F.; da Silva, E.L.; Borges, G.S.; Nunes, E.C.; Fett, R. Improvement of serum antioxidant status in humans after the acute intake of apple juices. *Nutr. Res.* **2012**, *32*, 229–232.
49. Wang, M.Y.; Lutfiyya, M.N.; Weidenbacher-Hoper, V.; Anderson, G.; Su, C.X.; West, B.J. Antioxidant activity of noni juice in heavy smokers. *Chem. Cent. J.* **2009**, *3*, doi:10.1186/1752-153X-3-13.
50. Wang, M.Y.; Peng, L.; Jensen, C.J.; Deng, S.; West, B.J. Noni juice reduces lipid peroxidation-derived DNA adducts in heavy smokers. *Food Sci. Nutr.* **2013**, *1*, 141–149.
51. Yuan, L.; Meng, L.; Ma, W.; Xiao, Z.; Zhu, X.; Feng, J.F.; Yu, H.; Xiao, R. Impact of apple and grape juice consumption on the antioxidant status in healthy subjects. *Int. J. Food Sci. Nutr.* **2011**, *62*, 844–850.
